# Understanding delusions

**DOI:** 10.4103/0972-6748.57851

**Published:** 2009

**Authors:** Chandra Kiran, Suprakash Chaudhury

**Affiliations:** Department of Psychiatry, Ranchi Institute of Neuropsychiatry and Allied Sciences, Kanke, Ranchi - 834 006, Jharkhand, India

**Keywords:** Delusions, Etiology, Psychopathology, Phenomenology

## Abstract

Delusion has always been a central topic for psychiatric research with regard to etiology, pathogenesis, diagnosis, treatment, and forensic relevance. The various theories and explanations for delusion formation are reviewed. The etiology, classification and management of delusions are briefly discussed. Recent advances in the field are reviewed.

“There is no delusional idea held by the mentally ill which cannot be exceeded in its absurdity by the conviction of fanatics, either individually or en masse”…Hoche

A delusion is a belief that is clearly false and that indicates an abnormality in the affected person’s content of thought. The false belief is not accounted for by the person’s cultural or religious background or his or her level of intelligence. The key feature of a delusion is the degree to which the person is convinced that the belief is true. A person with a delusion will hold firmly to the belief regardless of evidence to the contrary. Delusions can be difficult to distinguish from overvalued ideas, which are unreasonable ideas that a person holds, but the affected person has at least some level of doubt as to its truthfulness. A person with a delusion is absolutely convinced that the delusion is real. Delusions are a symptom of either a medical, neurological, or mental disorder. Delusions may be present in any of the following mental disorders: (1) Psychotic disorders, or disorders in which the affected person has a diminished or distorted sense of reality and cannot distinguish the real from the unreal, including schizophrenia, schizoaffective disorder, delusional disorder, schizophreniform disorder, shared psychotic disorder, brief psychotic disorder, and substance-induced psychotic disorder, (2) Bipolar disorder, (3) Major depressive disorder with psychotic features (4) Delirium, and (5) Dementia.

## HISTORY

The English word “*delude*” comes from Latin and implies playing or mocking, defrauding or cheating. The German equivalent *Wahn* is a whim, false opinion or fancy and makes no more comment than the English upon the subjective experience. The French equivalent, *delire* is more empathic; it implies the ploughshare jumping out of the furrow (lira), perhaps a similar metaphor to the ironical ‘unhinged’. Since time immemorial, delusion has been taken as the basic characteristic of madness. To be mad was to be deluded. What is delusion is indeed one of the basic questions of psychopathology. It would be a superficial and wrong answer to this question just to call a delusion a false belief which is held with incorrigible certainty. We may not hope to resolve this issue quickly with a definition. Delusion is a basic phenomenon. It is the primary task to get this into view. The subjective dimension within which delusion exists is to experience and think our reality (Jaspers, 1973). Whether we like it or not, this is the unavoidable field of tension in which research on delusions is situated: A tight, objectivity-oriented conceptualization on the one hand and the basic anthropological dimensions of subjectivity and interpersonality (i.e. human interdependence or “universal fraternity”) on the other hand. Even if one is skeptical about these “basic” aspects, Jaspers’ central idea should be kept in mind: Delusion is never a mere object which can be objectively detected and described, because it evolves and exists within subjective and interpersonal dimensions only, however “pathological” these dimensions may be. This reminds us of a central topic of psychiatric research: There are two fundamentally different approaches to research on complex mental phenomena, be they normal or pathological.

- The first approach—the “naturalistic” one—regards the complexity and heterogeneity of scientific means to study delusion as a temporary phenomenon, as the second-best solution. This solution, according to the naturalistic perspective, will only be used until a strictly empirical neuroscientific approach has progressed far enough to replace mentalistic vocabulary with a neurobiological one. In this view mental phenomena are identical with their neurobiological “basis”. In other words, mental events are not regarded as a distinct class of phenomena, either gradually or principally. “Eliminative materialism” is the most radical position in this context, which declares terms such as intention, willful action, individual values, personality or autonomy to be part of “folk psychology”. According to this approach, these terms may well be useful socially and on an every-day-life basis, but not scientifically, and they will be replaced, “eliminated”, by the language of neurobiology in the not too distant future.
- The second approach—the “phenomenological point of view” in Jaspers’ terms—departs from a person’s subjective experiences as the core issue of scientific studies on psychopathology. This does not, of course, exclude neurobiological research strategies at all, but it does insist on the scientific significance of the subjective dimension. Research into delusions is one of the most interesting examples of the importance of this methodological dichotomy. We will briefly review some of the major concepts of delusional thinking as they appeared from the 19^th^ century until today.

## DESCRIPTIVE PHENOMENOLOGICAL APPROACH

This approach to understanding delusions is a very influential one for psychiatrists. Jaspers’ book *General Psychopathology* marked a major step forwards in establishing psychopathology as a scientific discipline. Experiencing mental states by the patient and the understanding of this experience by the physician defined the central framework. However, in contrast to biological phenomena, mental events in Jaspers’ view can never be accessed directly, but only via the expressions of the person who experiences them. Phenomenology is the study of subjective experience. It is one’s empathic access or understanding of the patient’s experience. One enters into the other person’s experiences using the analogy of one’s own experience. Jaspers distinguishes between static understanding which “grasps particular psychic qualities and states as individually experienced” and genetic understanding which “grasps the emergence of one psychic event from another”. Phenomenology is static understanding. Phenomenology is one’s recreation of the patient’s experiences through “transferring into”, “empathizing with” or literally “feeling into” or “living with” the patient’s experiences. In this way one arrives at an “actualization, representation or bringing to mind” of the patient’s experience. “Phenomenology actualizes or represents the patient’s inner subjective experiences. We can only make a representation of them through an act of empathy or understanding” (Jaspers, 1963).

## THE CONCEPT OF FORM AND CONTENT

Following the theory of knowledge of the philosopher Immanuel Kant, Jaspers accepts that all experience or knowledge entails both an incoming sensation and an organizing concept. The former is matter or content, the latter is form. The empiricists (Locke, Berkeley& Hume) emphasized incoming sensation exclusively; the rationalists (Descartes and Leibniz) emphasized the organizing concept exclusively. Kant took a carefully considered middle course. All experience and knowledge entails the two stems of conceptual form and intuitive content. This will be crucial for Jaspers’ concept of delusion. In Kant’s words from his *Critique of Pure Reason*: “That in the appearance which corresponds to sensation I term its matter (or content) but that which so determines the manifold of appearance that it allows of being ordered in certain relations, I term the form of the appearance”. This is the philosophical origin of the concept of form and content within Jaspers’ psychopathology. The differing forms of psychopathological experience are the topic for phenomenology. In his early paper, *The Phenomenological Approach to Psychopathology*, Jaspers spells it out that “phenomenological definitions" relate to the “different forms of experience”: “From its beginnings, psychiatry has had to concern itself with delimiting and naming these different forms of experience; there could, of course, have been no advance at all without such phenomenological definitions”. This is the crucial point that Jaspers is at pains to make - that phenomenology is primarily concerned with form and that content is largely irrelevant: “Phenomenology only makes known to us the different forms in which all our experiences, all psychic reality, takes place; it does not teach us anything about the contents”. Then, in *General Psychopathology*, Jaspers becomes more explicit about the concept of form. Form is the mode or the manner in which we experience content: “Perceptions, ideas, judgments, feelings, drives, self-awareness, are all forms of psychic phenomena; they denote the particular mode of existence in which content is presented to us”. The same content can be presented in different forms. The two Kantian stems are thought of by Jaspers as subject and object. The subjective stem is the conceptual form imposed by the mind and the objective stem is the incoming content of intuition or sensation. As a content presenting in different forms, Jaspers gives the example of hypochondriasis: “In all psychic life there is subject and object. This objective element conceived in its widest sense we call psychic content and the mode (Art) in which the subject is presented with the object (be it a perception, a mental image or thought) we call the form. Thus, hypochondriacal contents, whether provided by voices, compulsive ideas, overvalued ideas or delusional ideas, remain identifiable as content” (Jaspers, 1963).

## THE FORMS OF BELIEF

Jaspers distinguishes four forms of beliefs, i.e. four distinct modes or ways in which beliefs can be presented to consciousness. These are normal belief, overvalued idea, delusion-like idea and primary delusion. In the English literature, the delusion-like idea is usually known as the secondary delusion but Jaspers himself does not use this term. The English literature tends either to split these four forms into two pairs on the basis that normal belief and overvalued idea both occur in ‘normal’ psychic life while delusion-like idea and primary delusion always reflect an ‘abnormal’ mental state, or to split off the primary delusion on the grounds that the other three are understandable while the primary delusion is not. Both Cutting and Sims refer to the first distinction while only Sims notes the second (Cutting, 1985; Sims, 1988). This first distinction emphasizes whether the belief is delusional in nature or merely overvalued. Sims (1988), in *Symptoms in the Mind*, appeals to Jaspers and gives the following criteria for delusion: (a) They are held with unusual conviction. (b) They are not amenable to logic. (c) The absurdity or erroneousness of their content is manifest to other people. Cutting (1985), in his *The Psychology of Schizophrenia*, gives an almost identical definition, again with an appeal to Jaspers. These three features (extraordinary conviction and certainty, imperviousness or incorrigibility and impossible content) are the ones usually taken to distinguish delusion from other beliefs. Sims and Cutting are correct that Jaspers does say exactly that of delusions: (a) They are held with an extraordinary conviction, with an incomparable subjective certainty. (b)There is imperviousness to other experiences and to compelling counter-argument. (c) Their content is impossible. What Sims and Cutting miss is that Jaspers says that these are merely the ‘external characteristics’ of delusion. They are characteristic of delusion but they fail to account for the essential differences between delusion and other forms of belief. In fact, Jaspers dismisses these criteria in the first paragraph of his account: “To say simply that a delusion is a mistaken idea which is firmly held by the patient and which cannot be corrected gives only a superficial and incorrect answer to the problem” (Jaspers, 1963).

It is easy to demonstrate the inadequacy of these criteria. Imagine two politicians with opposing beliefs. Both hold views with an ‘extraordinary conviction’ and ‘an incomparable subjective certainty’. Both show a very definite ‘imperviousness to other experiences and to compelling counter-argument’. For each, the judgments of the other are ‘false’, and ‘the content impossible’. Obviously, neither is deluded. Both are expounding views which are highly valued, or perhaps overvalued, but which fulfill the above ‘external characteristics’ of delusional belief. Sims’ and Cutting’s criteria must be deemed inadequate to distinguish delusions from other firmly held beliefs and the expression “held with delusion-like intensity” as an essential criterion for delusion is therefore nonsense. Plenty of other beliefs besides delusions are “held with delusion-like intensity”. Even the truth or falsehood of the content of a belief is inadequate to distinguish a delusion. Jaspers is quick to point out that the content of some delusions is true, e.g. in pathological jealousy, where the wife is having an affair but the patient is right for the wrong reasons and is therefore still deluded (Jaspers, 1963). With some awareness of the above problems, Sims adds the second distinction based on understanding. *A delusion, unlike an overvalued idea, ‘is not understandable’ in terms of the patient’s cultural and educational background although the secondary delusion (or delusion-like idea) is understandable with the addition of some other psychopathological event such as hallucination or abnormal mood*. The standard preoccupation remains whether any belief is delusional or merely overvalued.

## DELUSION-LIKE OR OVERVALUED IDEAS

This standard view has difficulties with a wide variety of strange beliefs, some of which are examined below. Are they delusional or merely overvalued? On the above three ‘external characteristics’ plus understandability, they can certainly look very much like delusion-like ideas but the logical implication that this means a diagnosis of ‘psychosis’ is unacceptable and requires some very deft intellectual footwork to avoid. Are not the disordered beliefs of body image in anorexia derived from (understandable in terms of) the fear of gaining weight and the preoccupation with food and, if so, why do we not consider them to be delusion-like rather than overvalued? Why do we not consider the catastrophe beliefs in severe obsessional states to be delusion-like? Catastrophe beliefs are closely linked to the underlying compulsions and such patients do often believe that a failure to carry out the ritual will result in some dreadful catastrophe. Beck and his associates have described a range of abnormal cognitions consequent on depressive and anxiety states (Beck *et al*, 1979). We would regard many of these depressed patients as neurotic rather than psychotic depressives but nevertheless they have very compelling automatic thoughts and negative schemata of failure, hopelessness, and helplessness, clearly linked to (understandable in terms of) their mood disorders. Can the beliefs of miscast gender in transsexualism be passed off glibly as overvalued idea when personality trait makes the belief understandable?

A further example can be found in pathological gambling. Although the experienced gambler knows that the casino game is rigged against him and that in the long term the house must win, he continues to believe in his own luck. Wagenaar (1988) found a web of illogical cognitions in compulsive gamblers. Many were magical in quality and some actually made it more likely that the gambler would lose. At roulette, a game of chance entirely, players had a strong tendency to leave their chips on a winning number on the grounds that it was lucky. When they lost they tended to put their chips on numbers that have not yet won. Backing a number adjacent to, or arithmetically related to, the winner meant their luck was returning. Wagenaar points out that few numbers are not related either by proximity or by arithmetic so that the gambler can always ‘delude’ himself that his luck is rising and that a win is imminent. Some gamblers had an elaborate system, provoking the old telegram “System perfected, send more money”. Many gamblers believed that chance and luck were not just abstract ideas but were causal forces which were open to manipulation. Roulette is the closest thing to random numbers outside a computer but many of Wagenaar’s gamblers had developed beliefs which were magical in nature, defied the laws of mathematics and, in some cases, were actually helping them to lose. These magical beliefs are derived from (are understandable in terms of) the compulsion, the arousal and the excitement of pathological gambling.

Walker (1991) has proposed that: (1) A number of ideas both inside and outside psychopathology have at least a *prima facie* case to be considered delusion-like (they fulfill the three ‘external characteristics’ and they are understandable in terms of unusual, if not psychopathological, experiences). (2) an intellectual sleight of hand is often in operation in the distinction of overvalued and delusion-like. If we intend to make a ‘psychotic’ diagnosis, then the belief is delusion-like; if we intend to make a ‘non-psychotic’ diagnosis, then the belief is overvalued. The phenomenology is molded to fit. (3) The third aim is to suggest, with Jaspers, that, as both an overvalued idea and a delusion-like idea are understandable (the overvalued idea in terms of the personality and life experiences and the delusion-like idea in terms of the same plus some other psychopathological event) there is little to be gained by their distinction. Jaspers solves this problem neatly by shifting the whole emphasis. For him, the important distinction is not between overvalued idea and delusion-like idea but rather between delusion-like idea and primary delusion. Jaspers’ terminology has importance for his account. Only the primary delusion is a ‘delusional idea proper’ for him and the delusion-like idea is, as its name suggests, not a true delusion but merely delusion-like. Jaspers, therefore, makes no real distinction between the overvalued idea and the delusion-like idea. There are several occasions when he simply equates the two. For example, in Jaspers (1963): “Exhaustion may help to develop a long-prepared delusion of reference (an overvalued idea)”. “Melancholia. In this state the overvalued or compulsive depressive ideas become delusion-like”. “Mood-states, wishes and drives give rise to delusion-like ideas (overvalued ideas) which arise in more or less understandable fashion from them”.

## THE ESSENTIAL CHARACTERISTICS OF DELUSION

Jaspers’ solution to the problem of delusion is as follows: “If we want to get behind these mere external characteristics of delusion into the psychological nature of delusion, we must distinguish the original experience from the *judgment based* on it, i.e. the delusional contents as presented data from the fixed judgment which is then merely reproduced, disputed, dissimulated as occasion demands”. The essential criterion distinguishing the different forms of belief lies not in their conviction and certainty, not in their incorrigibility and not in their impossible content but in their origins within the patient’s experience. Jaspers goes on: “We can then distinguish two large groups of delusion according to their origin: one group *emerges understandably* from preceding affects, from shattering, mortifying, guilt-provoking or other experiences, from false-perception or from the experience of derealisation in states of altered consciousness, etc. The other group is for us psychologically irreducible; phenomenologically it is something final. We give the term *delusion-like* to the first group; the latter we term *delusions proper*”. Thus, the essential distinguishing factor within the four forms of belief is the concept of understanding. One can understand the evolution or development of the normal belief and the overvalued idea from the personality and its life events. One can understand the delusion-like idea from personality, life events and from some other psychopathological experience but the primary delusion is something new, irreducible and non-understandable. The primary delusion is of paramount importance for Jaspers. Including the above distinction of a lack of understandability, the primary delusion differs in three ways from the other three forms of belief: (a) The primary delusion is unmediated by thought. (b) The primary delusion is ununderstandable. (c) The primary delusion implies a change in ‘the totality of understandable connections’ which is personality.

## PRIMARY DELUSION AS AN UNMITTELBAR PHENOMENON

Cutting across the whole of phenomenology is the distinction between ‘direct’ or ‘immediate’ (*unmittelbar*; literally ‘unmediated’) experiences and experiences which are the result of reflection or thought and which are ‘indirect’ (*gedanklich vermitteltes*: Literally ‘mediated by thought’). This distinction of phenomena which are ‘unmediated’ and those which are ‘mediated by thought’ ‘overlaps’ all other divisions. Jaspers does try to clarify what he has in mind by this distinction. Immediate, direct or unmediated experiences he describes as experiences which are ‘elementary’ and ‘irreducible’. In contrast, experiences which are mediated by thought he describes as “developed, evolved, based on thinking and working through”; that is they are the product of reflection. The distinction is crucial: We have to distinguish between immediate certainty of reality and reality judgment. Reality-judgment is the result of a thoughtful digestion of direct experiences” (Jaspers, 1963). The primary delusion is a direct, unmediated phenomenon; the delusion-like idea is reflective or mediated by thought: “The primary delusional experience is the *direct, unmediated* intrusive knowledge of meaning. not considered interpretations but meaning *directly* experienced”. On the other hand: “Delusion-like ideas. emerge understandably from other psychic events and can be traced back psychologically to certain affects, drives, desires and fears”. Jaspers gives some further examples of the distinction [Table 1]. The primary delusion is a direct, immediate or unmediated phenomenon while the other three forms of belief are all mediated by thought. That is, normal beliefs, overvalued ideas and delusion-like ideas are all reflective, considered interpretations. In fact, the primary delusion is essentially not a belief or judgment at all but rather an experience. Jaspers writes exactly that “Phenomenologically it is an experience”. The German is primare *Wahnerlebnis* - primary delusional experience.

**Table 1:** Jaspers’ distinction between unmediated or direct and mediated or reflective phenomena

Unmediated, direct experience	Thought-mediated experience
Delusional idea proper	Ordinary mistake
Concrete awareness (the sense of presence)	The ‘as-if’ experience
True hallucination	A fantastic image projecting itself illusively in space
A melancholic state	Neurotic depression as a result of an unpleasant event
The experience of one’s own double	The feeling as if there are “two psyches in my breast”
An instinctual drive	A simple wish
The urge to move	Understandable motor discharge of feelings

Primary delusion is the experience of delusional meaning. The experience of meaning (*Bedeutung*) is implicit in all perception and it is the distortion of this implicit meaning which is the primary delusional experience. Jaspers begins with examples from mundane perceptions: “All thinking is thinking about meanings. Perceptions are never mechanical responses to sense stimuli; there is always at the same time a perception of meaning. A house is there for people to inhabit. If I see a knife, I see directly, immediately a tool for cutting. We may not be explicitly conscious of the meanings we make when we perceive but nevertheless they are always present.” Jaspers goes on: “Now, the primary delusional experience is analogous to this seeing of meanings. The awareness of meaning undergoes a radical transformation. The direct or immediate, intrusive knowledge of meaning is the primary delusional experience. These are not considered interpretations but direct experiences of meaning while perception itself remains normal and unchanged. All primary delusional experience is an experience of meanings”.

The meaning ‘for people to inhabit’ is implicit in the perception of a ‘house’. The meaning ‘for cutting’ is implicit in the perception of a ‘tool’. In exactly the same way, the delusional meaning is implicit in the primary delusional experience. Examples of primary delusions will help clarify Jaspers’ meaning: “Suddenly things seem to mean something quite different. The patient sees people in uniform in the street; they are Spanish soldiers. There are other uniforms; they are Turkish soldiers. Then a man in a brown jacket is seen a few steps away. He is the dead Archduke who has resurrected. Two people in raincoats are Schiller and Goethe. [and from another patient]: In the morning I ran away; as I went across the square the clock was suddenly upside down; it had stopped upside down. I thought it was working on the other side; just then I thought the world was going to end; on the last day everything stops; then I saw a lot of soldiers on the street; when I came close, one moved away; ah, I thought, they are going to make a report; they know when you are a “wanted” person; they kept looking at me; I really thought the world was turning round me. In the afternoon the sun did not seem to be shining when my thoughts were bad but came back when they were good. Then I thought cars were going the wrong way; when a car passed me I did not hear it. I thought there must be rubber underneath; large Lorries did not rattle along anymore; as soon as a car approached, I seemed to send out something that brought it to a halt. I referred everything to myself as if it were made for me people did not look at me, as though they wanted to say I was altogether too awful to look at”. For Jaspers, these two patients, and especially the second, are facing a shower of new primary delusional meanings.

Kurt Schneider’s impetus from about 1925 onwards was to reformulate clinical psychopathology on a descriptive basis, avoiding interpretation and speculation wherever possible. It remained in accordance with Jaspers’ ideas of psychopathology; however, Schneider considered it important not to return to the elementary concept of association psychology, but to keep the clinical and biographical context in mind (Schneider, 1980). Schneider mainly dealt with delusions through their formal structure. He, too, was in search of a criterion that could differentiate reliably between “delusion proper” and “delusion-like phenomena”, and such a criterion, in his view, was “delusional experience” (Wahnwahrnehmung), which was defined as a two-step process: The sensory input is correct, whereas its interpretation is delusional. The patient, for example, sees a dark cloud in the sky, which, for him, is proof, beyond doubt, that he will die the day after. This, in Kurt Schneider’s view, is delusional in a narrow sense. Unless an organic lesion of the central nervous system can be identified, he regarded such an experience as a “first rank symptom” of schizophrenia.

## STRUCTURAL DYNAMIC APPROACH

The German psychiatrist and psychopathologist Werner Janzarik developed his theory of structural dynamics beginning in the 1950s. It is an interesting and underestimated approach to the understanding of psychotic disorders, beyond mere operationalism and beyond psychoanalytical interpretation. In mental life, healthy or disordered, Janzarik differentiated between structural components that are rather firm and longstanding, such as basic ideas and values, from their dynamic qualities, which mainly address the affective field. In healthy persons, the dynamic aspect is linked to certain structural components, which may have genetic or psychological origins or may just result from a learning process. In psychosis, including many delusional states, however, these dynamic forces, not being sufficiently integrated into the structural components, will show a “derailment”, clinically presenting as restriction—the depressive pole, expansion—the manic pole, or instability (Unstetigkeit)—the acute psychotic pole. In the latter case, there will often appear what is called an increasingly “impressive” way of experiencing. This means that in the patient’s perspective many, if not all perceptions, even those of minor or no importance for that person, gain high and embarrassing personal significance, albeit in an odd, vague (”impressive”) manner. Klaus Conrad (1958) gave a masterful description of this psychopathological phenomenon in his book on *Beginning Schizophrenia*. He argued that sensory input will be subjectively altered and will become symbolical, frightening, or even threatening. The psychotic person will often have the impression of ideas or experiences being forced upon him or her by an external power. This will clinically be described as a delusional syndrome.

## ANTHROPOLOGICAL AND “DASEINSANALYTICAL” APPROACH

Binswanger says that one must deal with human existence as a whole in order to understand its particular abnormalities. Delusion for Binswanger is a pathological type of world design. World design is a term which reflects the organization of all the conscious and unconscious attitudes of a human being towards all that is sensible. Minkowski attempts to characterize mental disorder as some single fundamental disturbance (trouble generator) and he thinks that all such disturbances are spatiotemporal in nature; by this he means that the patient with a delusion of persecution is no longer able to perceive the chance nature of all that happens around him owing to a feeling of restriction of freedom and movement (the spatiotemporal disturbance), and so refers it all to himself; thus in the delusion of persecution what the patient wants is not a feeling of benevolence towards him but a feeling of ease and freedom. Rumke maintains that delusion is a product of an ill, not a normal person. He offers as proof that after their recovery patients claim they did not mean exactly what they said. He also believes that delusion is a secondary and less important phenomenon, and that what is of real interest to the psychiatrist is the inner attitude of the patient, his world design and his way of thinking, even though, as he states, phenomenology of this kind will never teach us to explain the illness, it only puts us in a position to understand it. Kronfeld’s view can best be summarized as follows: A delusion is the result of the failure of the “objectifying act” because of the strength of the “intentional act.” By “objectifying act” is meant the exercise of man’s ability to be aware of his own intention and action, and by “intentional act” is meant the exercise of man’s ability to wish, desire and imagine some particular action. The strength of this intentional act may become so great that the ego fails to objectify it, *i.e*., to identify it correctly as a wish, and thus a delusion is established. Put simply, Kronfeld says that the delusional patient cannot distinguish between phantasy and reality; this has some conceptual similarity to the notion of projection: One does not recognize one’s own ideas as one’s own and attributes them to the external and objective environment. The anthropological approach and that of Daseinsanalyse considers the problem of delusions with regard to their specific relevance for the whole life of the deluded person. The central idea here is that within an existential crisis of the deluded person a delusion can serve as a kind of coping or problem-solving—albeit a “pathological” one from the perspective of others. Of course, this way of resolving the crisis itself creates more problems, and is even harmful, especially to communication with others. This is nevertheless a lesser evil for the sufferer, because it can allow a new stability of mental state, even though pathological. Here, a delusion (and psychosis in general) is understood as a very specific human way of “being in the world”, the roots of which lie in a basic disturbance of interpersonal communication.

## BIOGRAPHICAL APPROACH

The period of “romantic psychiatry”, which had a significant influence on the development of European psychiatry at least in the first decades of the 19^th^ century, focused on complex biographical and emotional aspects of human life more than on the rationalistic perspective, which, in turn, had been the central point of reference during the period of enlightenment in the 18^th^ century (Steinberg, 2004). This framework of romanticism was nearly swept away around 1850 by a naturalistic attitude, which was allied to the natural sciences and biologically oriented general medicine and psychiatry, which became more and more successful. Rather than going into detail on this specific issue, I want to address the re-discovery of the biographical approach to delusions in the early 20^th^ century. Early in the 20^th^ century two influential psychiatrists, Robert Gaupp and Ernst Kretschmer, focused on the correlation between biography and personality traits of people later diagnosed as deluded. Kretschmer coined the term “sensitive delusion of reference” (sensitiver Beziehungswahn). The main hypothesis was that vulnerable and anancastic personality traits in combination with real and repeated insults will first lead to a dysphoric and suspicious attitude, and then, if no solution is found, to delusion-like ideas and, finally, to a delusion proper. In contrast to the ideas of early psychoanalysis, this approach did not claim to explain the genesis of a delusion in the sense of causality, but to identify typical patterns of situations and conditions that lead to delusional states. This explicitly included biological factors, at that time often called “constitutional”. Kretschmer spoke of the need for a “multidimensional psychiatry”—a very modern concept indeed. The case that represents this approach most prominently is that of Ernst Wagner (1874-1938). He was a teacher, living with his family (his wife and four children) in Degerloch next to Stuttgart in southern Germany. In the night from 3 to 4 September 1913, he killed all five members of his family while they were sleeping and later shot or wounded at least 20 other persons and set fire to several houses. He was examined for forensic purposes by Robert Gaupp, who found him not responsible for his deeds because of the chronic development of a delusional disorder, with the background of having both sensitive personality traits and distressing life events. Wagner was not sent to jail, but remained in several psychiatric hospitals for decades, where he began to write dramas and novels.

## PSYCHOANALYTICAL APPROACH

For Freud and many of his early pupils, delusions—like the majority of psychopathological symptoms—were the result of a conflict between psychological agencies, the id, ego, and super-ego. Delusion, briefly stated, is seen as a personal unconscious inner state or conflict which is turned outwards and attributed to the external world. He considered that latent homosexual tendencies especially formed the basis of paranoid delusions. Later, psychoanalytical authors gave up this very narrow hypothesis and suggested that delusions might be a compensation for any—i.e. not necessarily sexuality-related—kind of mental weakness, e.g. lack of self-confidence, chronic anxiety or identity disturbances. This concept in a way resembles Alfred Adler’s theory of individual psychology, in which the consequences of personal failures or shortcomings play a major role in the etiology and pathogenesis of (neurotic) mental disorders (Adler, 1997). The best known example for the application of the above mentioned psychoanalytical arguments in the debate on delusion is Freud’s paper on the Schreber case.

## NEUROBIOLOGICAL APPROACH

There still is no comprehensive neurobiological theory of delusion formation or maintenance, although various empirical, conceptual and speculative arguments have been proposed, often resulting from the discussion of psychotic states occurring during neurological disorders (Munro, 1994). In recent decades there has been significant progress in psychopharmacology, psychiatric genetics and functional neuroimaging in the study of psychotic and affective disorders. The problem remains, however, that most neurobiological studies have not addressed delusions per se, nor delusional disorder/paranoia, due to its rarity. Rather, they tend to be about schizophrenic or, worse, “psychotic” disorders in all their heterogeneity. These psychoses may or may not have had delusional features. So, all the neurobiological hypotheses that were suggested in connection with delusional syndromes must be read with the caveat that they might—at least partly—relate more to psychosis than to delusion, e.g. the hypotheses of hyperdopaminergic activity, functional disconnection of frontal and temporal brain areas, or disturbed basal information processing, as detectable by evoked potential techniques. The clinical efficacy of antipsychotics in acutely psychotic patients with delusional and hallucinatory syndromes is an argument in favor of the hypothesis of dopaminergic hyperactivity in mesolimbic and mesocortical circuits, since these agents have in common their dopamine antagonistic properties. As for delusions, however, this efficacy is typically limited to acute or subacute states, whereas chronic delusions, and especially the rare condition of paranoia, often, although not invariably, prove resistant to antipsychotic (and other biological and psychotherapeutic) treatments. A hypothesis proposed by Spitzer (1995) combines the aspect of disturbed dopaminergic neurotransmission in deluded patients with the concept of neural networks derived from computational science. On the basis of replicated findings from word association studies (”semantic priming paradigm”), he suggests that elevated dopaminergic transmission will result in an increased signal-noise difference in the neural network. In computer simulation models, the artificial net will show properties that—in a far-reaching conclusion by Spitzer—resemble clinical features of deluded patients, e.g. the tendency to relate any experience, however irrelevant it may objectively be, to the patient’s personal situation, often in a negative or even threatening way.

## ANALYTICAL PHILOSOPHY OF MIND/LINGUISTIC APPROACH

In recent philosophical literature, there is an interesting line of thought concerning the qualitative status of subjective experiences that is important for the psychiatrist. The meaning of “qualitative” here is the specific quality of a certain experience, for example the experience of color or pain. This is usually called the “qualia-problem”. The question is what precisely makes the difference between a statement of internal experience (e.g., “I like that rich red color”) and a statement about the outer world (e.g., “It is raining”). An important difference is that utterances about one’s own mental states are not subject to external validation and there is little expectation of testing them, whereas statements about the outer world are always verifiable and subject to corrections, whether by observation or superior rational arguments by another person. To make this central issue more concrete, the statements, “I have a headache”, “I am sad”, and, “I am angry”, cannot be “corrected” by any argument by another person. The property “incorrigibility”—at least since Jaspers’ writings—also constitute a prominent criterion of delusional states. Spitzer (1990) applied this formal argument to delusional statements and came to the conclusion that we should identify delusion whenever a person speaks about the outer world with the same high degree of subjective certainty that is usually only observed in utterances about one’s “inner” experiences—i.e. with the quality of “incorrigibility”. For example, if a paranoid person says that he or she is being observed by the secret service all day, this statement, if delusional, would have the same ‘incorrigible’ degree of subjective certainty as the sentence, “I am sad”.

## HALLUCINATIONS

A delusion might be an attempt at explaining a hallucinatory experience. Wernicke called such a delusion, delusion of explanation. However, even the early description by Lasegue in 1852 of delusions of persecution and of their common association with auditory hallucinations never firmly stated the temporal relationship between delusions and hallucinations. We cannot call upon any established knowledge in the field of study of hallucinations to help answer the question. French psychiatry does distinguish two types of hallucinations, one of which is, one might hold, more like a delusion than hallucination. The two types are the true hallucination with full impression of the external nature of the sensation and the so-called mental hallucination where there is no impression of the external nature of the sensation, only a belief that one has seen something, or very commonly, that one has heard voices or noises or persons talking to one. The phenomenon of mental hallucination probably deserves a place amongst the other phenomena of delusion and hallucination.

## DE CLERAMBAULT’S AUTOMATISMS

The role of the hallucinatory types of experience is better discussed together with all the other so-called automatisms. De Clerambault holds that delusions are the reactions of an abnormal personality to automatisms. Briefly, his theory is an anatomical hypothesis that systematized chronic hallucinatory psychosis is based on anatomical processes in the brain due to infections, lesions, toxins, traumata or sclerosis. These anatomical insults produce mental automatisms which mark the beginning of the psychosis. Contrary to prevalent beliefs de Clerambault maintained that at the beginning these automatisms were neutral in feeling tone. The patient tended to be puzzled by them but they were neither pleasant nor unpleasant. De Clerambault also described these automatisms as non-sensory in character, to distinguish them from hallucinations [Table 2]. A patient assailed by such automatisms may attempt to explain them as intentional and produce delusions such as delusions of influence, possession, persecution and so on. De Clerambault’s theoretical notions regarding the causation of chronic hallucinatory psychosis have been subjected to criticism. In the absence of published studies of the frequency and nature of the relationship between the automatisms and delusional states, the automatisms remain as hypothetical causes of delusions.

**Table 2:** De Clerambault’s automatisms (Derived from Baruk, 1959)

Mental	Sensory	Motor
Mental hallucinations	Bizarre	Kinesthetic
Constant parade of memories	Sensations	Sensations
ldeorrhea-.-gushing out of random ideas	Pricks	Involutionary
Strangeness of things	Currents	Gestures
Feelings of familiarity	Pulverized corpuscles	Levitation
False recognition		
Disappearance of thought		
Forgetting of thought		
Emptiness of thought		
Arrest of thought		
Feelings of perplexity		
Feelings of doubt		
Substitution of thoughts		
Disturbances of attention		
Affective, emotional, volitional automatisms		
Loss of visual memories		

## PERCEPTIONAL APPROACH

As Maher (1974) suggested, a delusion is—contrary to the classical position—not a cognitive disturbance, especially leading to flawed conclusions from correctly perceived sensory input, but a normal cognitive reaction to unexpected, strange mental events, especially perceptions. In early stages of delusional or, more generally, psychotic disorders the patient may register distressing alterations in sensory qualities; e.g., things seem bigger or smaller than usual, or look, feel or smell different. Such deeply worrying strangeness of experiences is regarded as the starting point of a development leading from suspiciousness to vague paranoid ideation and, finally, to systematized delusions. These experiences may be partly explained or at least made less frightening by the construction of a theoretical background of someone “who does all this deliberately” on the grounds of certain motives, be they known to the patient or not. This position, of course, marks a sharp contrast to Kurt Schneider’s view of “delusional experience”.

## ATTRIBUTIONAL AND COGNITIVE PSYCHOLOGY APPROACH

Since the 1990s there has been an increase in psychological research on cognitive processes in deluded patients. In this line of thought, the traditional assumption of undisturbed cognitive functions in delusional disorder, i.e. pathological content on the basis of normal form of thought, was questioned. In order to come closer to delusion-related phenomena themselves—as compared to the much broader psychosis-related phenomena—a number of studies compared patients with and without delusional ideation. Such a process also led to a number of interesting therapeutic implications. Three approaches are worthy of mention.

- Decision-making paradigm: Several groups found that in simple, affectively neutral decision-making paradigms, a deluded person needs less information to arrive at a definite decision than persons without a delusion or people with a depressive disorder. The latter needed significantly more information. With regard to delusions, this phenomenon was called “jumping to conclusions” and was interpreted as an argument for disturbed cognitive processes in the case of (persecutory) delusion (Garety& Freeman, 1999).
- Attribution psychology: A number of research groups confirmed the finding that, in comparison to healthy persons, deluded patients tend to attribute negative events or situations more often to other people or to external circumstances and not to themselves. This is also true for topics that have nothing to do with the actual delusional theme. For clinicians having had experience with paranoid patients, this is not a surprising finding, but it becomes interesting when regarded as an argument in favor of stable pathological patterns in the social cognition of deluded persons. Recently, this path has reached beyond the attributional perspective itself and encompasses cognitive models of delusional thinking in general, sometimes with a strong neurobiological impact (Blackwood *et al*., 2001).
- Theory of mind: According to Frith& Frith (1999), patients with paranoid schizophrenia suffer from a deficit in understanding correctly what others think about the patient and what their future attitudes or actions towards the patient might be. This phenomenon is well known from autism research, and is often called “theory of mind deficit”. It is the reduced ability to form a valid hypothesis about another person’s state of mind with regard to oneself. Paranoid or, more generally speaking, delusional ideation in this view is a result of disturbed cognitive and social metarepresentation.

## DEFINITION OF DELUSION

There can be no phenomenological definition of delusion, because the patient is likely to hold this belief with the same conviction and intensity as he holds other non-delusional beliefs about himself; or as anyone else holds intensely personal non-delusional beliefs. Subjectively, a delusion is simply a belief, notion or idea.

- Kraepelin in the ninth edition of his Textbook defined delusional ideas as pathologically derived errors, not amenable to correction by logical proof to the contrary.
- As per Stoddart, a delusion is a judgment which cannot be accepted by people of the same class, education, race and period of life as the person who experiences it.
- Jaspers (1959) regarded a delusion as a perverted view of reality, incorrigibly held, having three components:
  1. They are held with unusual conviction
  2. They are not amenable to logic
  3. The absurdity or erroneousness of their content is manifest to other people.
- Hamilton (1978) defined delusion as ’A false, unshakeable belief which arises from internal morbid processes. It is easily recognizable when it is out of keeping with the person’s educational and cultural background.’
- According to Sims (2003), a delusion is a false, unshakeable idea or belief which is out of keeping with the patient’s educational, cultural and social background; it is held with extraordinary conviction and subjective certainty.
- In the Diagnostic and Statistical Manual of Mental Disorders, a delusion is defined as: A false belief based on incorrect inference about external reality that is firmly sustained despite what almost everybody else believes and despite what constitutes incontrovertible and obvious proof or evidence to the contrary. The belief is not one ordinarily accepted by other members of the person’s culture or subculture (e.g. it is not an article of religious faith).

The fact that a delusion is false makes it easy to recognize but this is not its essential quality. A very common delusion among married persons is that their spouses are unfaithful to them. In the nature of things, some of these spouses will indeed have been unfaithful; the delusion will therefore be true, but only by coincidence (Casey& Kelly, 2008).

Kendler *et al*., (1983) have proposed several poorly correlated vectors of delusional severity:

1. Conviction: The degree to which the patient is convinced of the reality of the delusional beliefs.
2. Extension: The degree to which the delusional belief involves areas of the patient’s life.
3. Bizarreness: The degree to which the delusional belief departs from culturally determined consensual reality.
4. Disorganization: The degree to which the delusional beliefs are internally consistent, logical and systematized.
5. Pressure: The degree to which the patient is preoccupied and concerned with the expressed delusional beliefs.
6. Affective response: The degree to which the patient’s emotions are involved with such beliefs.
7. Deviant behavior resulting from delusions: Patients sometimes, but not always, act upon their delusions.

## CLASSIFICATION

There is no recognized way of classifying delusions according to any phenomenological principles. Tables 3 and 4 gives the classification given by Cutting (1997).

**Table 3:** Phenomenological classification of delusions

Inexplicability	Primary/pure /true Secondary delusion-like idea
	Overvalued idea
Subverted mental function	Delusional perception
	Delusional notion
	Delusional memory
	Delusional awareness
	Delusional mood/atmosphere
Alleged psychological antecedent	Misinterpretative delusional state Confabulatory delusional state
	Hallucinatory delusional state
	Delusional misidentification (e.g. Capgras’ syndrome) Sensitive delusions of reference
	Folie a deux
Nosological status	Paranoia/ delusional disorder/ monodelusional disorder
	Delusional loving/ erotomania/ de Clerambault’s syndrome
	Monosymptomatic hypochondriacal psychosis
	Cotard’s syndrome/nihilistic delusional state
	Delusional depression
	Schizophrenia-like psychosis
Thematic content	Persecutory, reference, influence/control, jealousy, sin, poisoning, theft, pregnancy, grandiose, infestation, lycanthropy, etc.
Mode of misconstruing world	Misidentification
	Misclassification
	Misattribution
	Mis-substantiation

**Table 4:** Classification of delusions according to cause (Cutting 1997)

Diagnostic link	Dementia, delirium, schizophrenia, depressive psychosis, mania	
Purported mechanism	Antecedent phenomenological condition	
	(a) For primary delusions	End of the world experience Trema- fright Anomalous experience Feeling of conviction
	(b) For secondary delusions	Depressed mood Elated mood Hallucinations Illogical thinking
	Impasse in life with personality predisposition	
	(a) For primary delusions	Inner conflict Ontological meaning search
	(b) For secondary delusions	Personality disorder Inner conflict External conflict Existential dilemma Exaggerated cognitive bias
	Psychological deficit	
	(a) For primary delusions	Thought disorder Breakdown in Gestalt
	(b) For secondary delusions	Anxiety Stimulus overgeneralization Breakdown in response hierarchies Impaired attention Perceptual deficit Heightened consciousness Illogical thinking General cognitive integration failure
	Extra-mental events	
	Applicable to primary and secondary delusions	Brain disease Sensory deprivation Deafness

### Primary and secondary delusions

The term primary implies that delusion is not occurring in response to another psychopathological form such as mood disorder. According to Jaspers the core of primary delusion is that it is ultimately un-understandable. Secondary delusions are understandable when a detailed psychiatric history and examination is available. That is, they are understandable in terms of the patient’s mood state, to the circumstances of his life, to the beliefs of his peer group; and to his personality. A delusion, whether primary or secondary in nature, is based on delusional evidence: the reason the patient gives for holding his belief is like the belief itself, false, unacceptable and incorrigible. Gruhle (1915) considered that a primary delusion was a disturbance of symbolic meaning, not an alteration in sensory perception, apperception or intelligence. Wernicke (1906) formulated the concept of an autochthonous idea; an idea which is native to the soil, aboriginal, arising without external cause. The trouble with finding supposed autochthonous or primary delusions is that it can be disputed whether they are truly autochthonous. For this reason they are not considered of first rank in Schneider’s (1957) classification of symptoms.

### Types of primary delusions

Delusional mood/atmosphere; Delusional perception; Delusional memory; Delusional ideas; Delusional awareness.

#### Delusional mood

It is usually a strange, uncanny mood in which the environment appears to be changed in a threatening way but the significance of the change cannot be understood by the patient who is tense, anxious and bewildered. Finally, a delusion may crystallize out of this mood and with its appearance there is often a sense of relief.

#### Delusional perception

In this an abnormal significance, usually in the sense of self-reference, despite the absence of any emotional or logical reason, is attributed to normal perception. Jaspers delineated the concept of delusional percept; and Gruhle (1915) used this description to cover almost all delusions. Schneider(1949) considered the essence of delusional perception to be the abnormal significance attached to a real percept without any cause that is understandable in rational or emotional terms; it is self-referent, momentous, urgent, of overwhelming personal significance and of course false.

#### Delusional memory

This is the symptom when the patient recalls as remembered an event or idea that is clearly delusional in nature, that is, delusion is retrojected in time. These are sometimes called retrospective delusions.

#### Delusional ideas

They appear abruptly in the patient’s mind, are fully elaborated, and unheralded by any related thoughts.

#### Delusional awareness

Delusional awareness is an experience which is not sensory in nature, in which ideas or events take on an extreme vividness as if they had additional reality. Delusional significance is the second stage of the occurrence of delusional perception. Objects and persons are perceived normally, but take on a special significance which cannot be rationally explained by the patient. Fine distinctions are sometimes imposed upon the classification of primary delusions, but are more collector’s items than features of useful clinical significance.

## CONTENT OF DELUSIONS

Delusions are infinitely variable in their content but certain general characteristics commonly occur. It is determined by the emotional, social and cultural background of the patient. Common general themes include persecution, jealousy, love, grandiose, religious, nihilistic, hypochondriacal and several others.

### Delusion of persecution

It is the most frequent content of delusion. It was distinguished from other types of delusion and other forms of melancholia by Lasegue (1852). The interfering agent may be animate or inanimate, other people or machines; may be system, organizations or institutions rather than individuals. Sometimes the patient experiences persecution as a vague influence without knowing who is responsible. May occur in conditions like: Schizophrenia, Affective psychosis: Manic, Depressive type, and Organic states: Acute, chronic. Persecutory overvalued ideas are a prominent facet of the litiginous type of paranoid personality disorder.

### Delusion of infidelity

Described by Ey (1950) may be manifested as delusion, overvalued idea, depressive affect or anxiety state. Various terms have been used to describe abnormal, morbid or malignant jealousy. Kraeplin used the term ‘sexual jealousy’. Enoch and Trethowan (1979) have considered the demonstration of delusion of infidelity in distinguishing psychotic from other types.

Mullen (1997) has classified morbid jealousy with disorders of passion in which there is an overwhelming sense of entitlement and a conviction that others are abrogating their rights. The other two are the querulant who are indignant at infringements of rights and the erotomanic who are driven to assert their rights of love. Delusion of infidelity may occur without other psychotic symptoms. Such delusions are resistant to treatment and do not change with time. Delusions of jealousy are common with alcohol abuse, they may also occur in some organic states, and are often associated with impotence, e.g. the punch-drunk syndrome of boxers following multiple contra-coup contusion. Morbid jealousy arises with the belief that there is a threat to the exclusive possession of his wife, but this is just as likely to occur from conflicts inside himself, his own inability to love or his sexual interest directed towards someone else, as from changing circumstances in his environment or his wife’s behavior. Husbands or wives may show sexual jealousy, as may sexual cohabitees and homosexual pairs. Morbid jealousy makes a major contribution to the frequency of wife battering and is one of the commonest motivations for homicide.

### Delusions of love

Erotomania was described by Sir Alexander Morrison (1848) as being: Characterized by delusions the patient’s love is of sentimental kind, he is wholly occupied by the object of his adoration, whom if he approach it is with respect. The respect the fixed and permanent delusions attending erotomania sometimes prompt those laboring under it to destroy themselves or others, for though in general tranquil and peaceful, the patient sometimes becomes irritable, passionate and jealous. Erotomania is commoner in women than men and a variety has been called ‘old maids insanity’ by Hart (1921), in which persecutory delusions often develop. These have sometimes been classified as paranoia, rather than paranoid schizophrenia; these delusional symptoms sometimes occur in the context of manic-depressive psychosis. Trethowan (1967) demonstrated the social characteristics of erotomania, relating the patient’s previous difficulties in parental relationships to the present erotomania. A variation of erotomania was described by and retains the name of de Clerambault (1942). Typically, a woman believes a man, who is older and of higher social status than she, is in love with her.

### Grandiose delusions

In this the patient may believe himself to be a famous celebrity or to have supernatural powers. Expansive or grandiose delusional beliefs may extend to objects, so leading to delusion of invention. Grandiose and expansive delusions may also be part of fantastic hallucinosis, in which all forms of hallucinations occur.

### Religious delusions

The religious nature of the delusion is seen as a disorder of content dependent on the patient’s social background, interests and peer group. The form of the delusion is dictated by the nature of the illness. So religious delusions are not caused by excessive religious belief, nor by the wrongdoing which the patient attributes as cause, but they simply accentuate that when a person becomes mentally ill his delusions reflect, in their content, his predominant interests and concerns. Although common, they formed a higher proportion in the nineteenth century than in the twentieth century and are still prevalent in developing countries.

### Delusions of guilt and unworthiness

Initially the patient may be self-reproachful and self-critical which may ultimately lead to delusions of guilt and unworthiness, when the patients believe that they are bad or evil persons and have ruined their family. They may claim to have committed an unpardonable sin and insist that they will rot in hell for this. These are common in depressive illness, and may lead to suicide or homicide.

### Delusions of negation/nihilistic delusions

These are the reverse of grandiose delusions where oneself, objects or situations are expansive and enriched; there is also a perverse grandiosity about the nihilistic delusions themselves. Feelings of guilt and hypochondriacal ideas are developed to their most extreme, depressive form in nihilistic delusions.

Factors concerned in the germination of delusions:

1. Disorder of brain functioning
2. Background influences of temperament and personality
3. Maintenance of self-esteem
4. The role of affect
5. As a response to perceptual disturbance
6. As a response to depersonalization
7. Associated with cognitive overload.

Factors concerned in the maintenance of delusions:

1. The inertia of changing ideas and the need for consistency
2. Poverty of interpersonal communication
3. Aggressive behavior resulting from persecutory delusions provokes hostility
4. Delusions impair respect for and competence of the sufferer and promote compensatory delusional interpretation.

None of these factors are absolute but any or all may act synergistically to initiate and maintain delusion.

## STAGES OF DELUSION FORMATION

Conrad proposed five stages of which are involved in the formation of delusions:

1. Trema: Delusional mood representing a total change in the perception of the world
2. Apophany: A search for, and the finding of new meaning for psychological events
3. Anastrophy: Heightening of the psychosis
4. Consolidation: Forming of a new world or psychological set based on new meaning
5. Residuum: Eventual autistic state.

## THEORIES OF DELUSION FORMATION

### Psychodynamic theory

Freud (1911) proposed that delusion formation involving denial, contradiction and projection of repressed homosexual impulses that break out from unconscious.

### Delusions as explanations of experience

Binswanger& Minkowski (1930) proposed disordered experiences of space and time leading to imprisoned and controlled feelings. Later in 1942 de Clerambault, put forth the view that chronic delusions resulted from abnormal neurological events (infections, intoxications, lesions). Maher offered a cognitive account of delusions which emphasized disturbances of perception. He proposed that a delusional individual suffers from primary perceptual abnormalities, seeks an explanation which is then developed through normal cognitive mechanism, the explanation (i.e. the delusion) is derived by a process of reasoning that is entirely normal. Also, delusion is maintained in the same way as any other strong belief. These are further reinforced by anxiety reduction due to developing explanation for disturbing or puzzling experiences.

### von Domarus rule

He postulated that delusions in schizophrenia arise from faulty logical reasoning. The defect apparently consists of the assumption of the identity of two subjects on the ground of identical predicates (e.g. Lord Rama was a Hindu, I am a Hindu, and therefore I am Lord Rama).

### Learning theory

Learning theorists have tried to explain delusions in terms of avoidance response, arising specially from fear of interpersonal encounter.

### Luhmann’s system theory

Luhmann defines that information, message and understanding connects the social systems with the psychic ones. If the psychic system fails to recognize the message of information correctly or is unable to negotiate between understanding and misunderstanding message, it detaches itself from the social system to which it is normally closely connected. This detachment releases the possibility of unhindered autistic fulfillment of desires and uncontrolled fear may appear as delusions.

### Neuro-computational model

The cerebral cortex can be viewed as a computational surface that creates and maintains dynamic maps of important sensorimotor and higher level aspects of the organism and its environment, reflecting the organism’s experience. Acute delusions are the result of an increased activity of the euromodulators dopamine and norepinephrine. This not only leads to a state of anxiety, increased arousal and suspicion, but also to an increased signal to noise ratio in the activation of neural networks involved in higher order cognitive functions, leading to formation of acute delusions. Alteration in the neuromodulatory state not only causes the occurrence of unusual experiences but also modify neruroplasicity which influences the mechanism of long term changes. So chronic delusions may be maintained by a permanently increased neuromodulatory state, or by an extremely decreased noradrenergic neuromodulatory state (Black wood *et al*., 2001).

## THEORIES OF NEUROCOGNITIVE AND EMOTIONAL DYSFUNCTION

### Theory of mind

It refers to the capacity of attributing mental states such as intentions, knowledge, beliefs, thinking and willing to oneself as well as to others. Amongst other things this capacity allows us to predict the behavior of others. Frith postulated that paranoid syndromes exhibit a specific ToM deficit, e.g., delusions of reference can be explained, at least in good part, by the patients’ inability to put themselves in another person’s place and thus correctly assess their behavior and intentions. Thought insertion and ideations of control by others can be traced back to dysfunctional monitoring of one’s own intentions and actions. Hence, thoughts enter the patient’s consciousness without his or her awareness of any intention to initiate these thoughts. Since deluded patients in symptomatic remission performed as well as normal controls at ToM tasks, ToM deficits seem to be a state rather than a trait variable.

### The role of emotions

Delusions driven by underlying affect (mood congruent) may differ neurocognitively from those which have no such connection (mood incongruent). Thus, specific delusion-related autobiographical memory contents may be resistant to normal forgetting processes, and so can escalate into continuous biased recall of mood congruent memories and beliefs. Regarding threat and aversive response, identification of emotionally weighted stimuli relevant to delusions of persecution has been seen.

### Probabilistic reasoning bias

It assumes that the probability-based decision-making process in delusional individuals requires less information than that of healthy individuals, causing them to jump to conclusions, which is neither a function of impulsive decision-making nor a consequence of memory deficit. Kemp *et al*., pointed out that deluded patients are not deluded about everything and that there may be no global deficit in reasoning abilities. The findings in reasoning abilities in delusional patients are only subtle and one might question the strength of their causality in delusional thinking.

### Theory of attributional bias

Bentall and others proposed that negative events that could potentially threaten the self-esteem are attributed to others (externalized causal attribution) so as to avoid a discrepancy between the ideal self and the self that is as it is experienced. An extreme form of a self-serving attributional style should explain the formation of delusional beliefs, at least in cases where the delusional network is based on ideas of persecution, without any co-occurring perceptual or experiential anomaly. During the course of illness, the preferential encoding and recall of delusion-sensitive material can be assumed to continually reinforce and propagate the delusional belief.

### Multifactorial model

**Figure 1:**
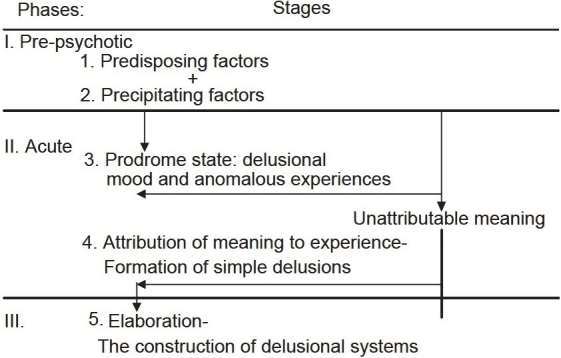
Roberts G. (1992) reviewed all concepts and gave the following general model of delusion formation

The emergence of symptoms assumed to depend upon an interaction between vulnerability and stress. Therefore the formation of delusion begins with a precipitator such as life event, stressful situations, drug use leading to arousal and sleep disturbance. This often occurs against the backdrop of long-term anxiety and depression. The arousal will initiate inner outer confusion causing anomalous experiences as voices, actions as unintended or perceptual anomalies which will turn on a drive for a search for meaning, leading to selection of explanation in the form of delusional belief [Figure 1].

### Neurobiological theories

The earlier works like Hartley (1834) suggested that vibration caused by brain lesion may match with vibrations associated with real perception. Ey (1952) believed delusion to be a sign of cerebral dysfunctions and Morselli listed the metabolic states for delusional pathogenesis. Jackson (1894) suggested pathogenesis of delusions due to combination of loss of functions of damaged part of brain. Cummings (1985) found that a wide variety of conditions can induce psychosis, particularly those that affect the limbic system, temporal lobe, caudate nucleus. He also noted that dopaminergic excess or reduced cholinergic activity also predispose to psychosis. He suggested that the common locus is limbic dysfunctions leading to inappropriate perception and paranoid delusion formation.

**Figure 2:**
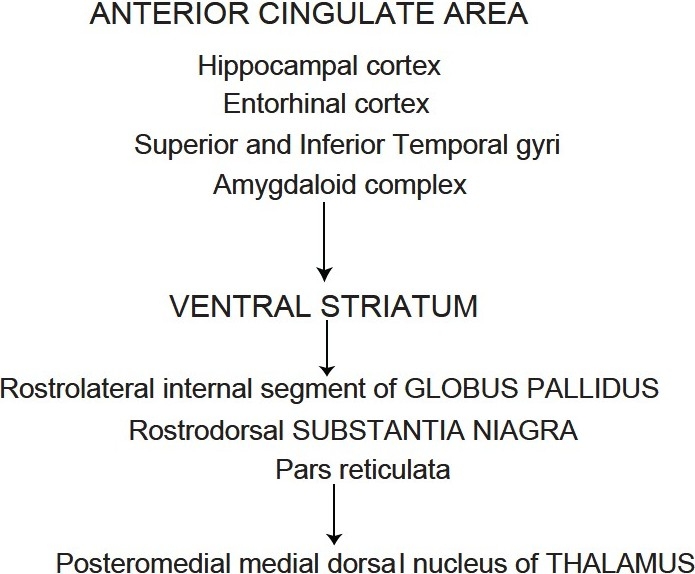
Alexander *et al*.’s (1986) proposed five structural functional loops

- Septo-hippocampal dysfunction model: The dysfunction leads to erroneous identification of neutral stimuli as important and judge expected as actual. Storage of erroneous information leads to delusion formation.
- Semantic memory dysfunction model: Delusions form due to inappropriate lying down of semantic memory and their recollections.
- Regional correlation with Alzheimer’s: Revealed a significant relationship between severity of delusional thought and the metabolic rates in three frontal regions. The study indicated that severity of delusions was associated with hypometabolism in additional prefrontal and anterior cingulate regions.
- Delusion of alien control has been linked with hyperactivation of the right inferior parietal lobule and cingulate gyrus, brain region important for visuospatial functions.
- Organic delusional disorders are more likely to be noted in extrapyramidal disorders involving the basal ganglia and thalamus and in limbic system disease. Alexander *et al*., (1986) proposed five structural functional loops. Any lesions, dysfunctions or derangements that affect any part of this loop can be expected to alter beliefs and emotional behavior [Figure 2].

## THE PERSISTENCE AND ELASTICITY OF DELUSIONS

Prediction error theories of delusion formation suggest that under the influence of inappropriate prediction error signal, possibly as a consequence of dopamine dysregulation, events that are insignificant and merely coincident seem to demand attention, feel important and relate to each other in meaningful ways. Delusions ultimately arise as a means of explaining these odd experiences (Kapur, 2003; Maher, 1974). The insight relief gained by arriving at an explanatory scheme leads to strong consolidation of the scheme in memory. In support of this view, aberrant prediction error signals during learning in patients with first-episode psychosis have been confirmed experimentally. Furthermore, the magnitude of aberrant prediction error signal correlated with delusion severity across a group of patients with first-episode psychosis. However, there are important characteristics of delusions that still demand explanation: Notably their persistence. Normal associations can extinguish if they prove erroneous, normal beliefs can be challenged and modified. But delusions are noteworthy for the fact that they remain even in the absence of support and in the face of strong contradictory evidence. We believe that this striking clinical phenomenon can be explained within the same framework by considering key findings from the animal learning literature, a literature that has been formerly invoked to explain chronic relapse to drug abuse; extinction and reconsolidation. If delusion formation may be explained in terms of associative learning then perhaps extinction may represent the process through which delusions are resolved. Extinction involves a decline in responding to a stimulus that has previously been a consistent predictor of a salient outcome. Prediction error is also central to extinction. It has been suggested that negative prediction error (a reduction in baseline firing rate of prediction error coding neurons) leads the organism to categorize the extinction situation as different from the original, reinforced, situation and it now learns not to expect the salient event in that situation. This learning focuses on contextual cues, allowing the animal to distinguish the newly non-reinforced context from the old, reinforced one. Extinction does not involve unlearning of the original association, but rather the formation of a new association between the absence of reinforcement and the extinction situation. Extinction experiences (the absence of expected reinforcement) invoke an inhibitory learning process which eventually overrides the original cue response in midbrain dopamine neurons. Individuals with psychosis do not learn well from these absent but expected events, nor do they consolidate the learning that does occur. But there is more to delusion maintenance than persistence in the absence of supportive evidence: delusions persist even when there is evidence that directly contradicts them. When confronted with counterfactual evidence, deluded individuals do not simply disregard the information. Rather, they may make further erroneous extrapolations and even incorporate the contradictory information into their belief. So, while delusions are fixed, they are also elastic and may incorporate new information without shifting their fundamental perspective.

## RESOLUTION OF DELUSION

Once a simple delusional belief is adopted with conviction, the subsequent course is very variable.

- Some patients have fleeting or brief delusional states, spontaneously remitting and returning to normal.
- Others respond well to standard treatment.
- Others elaborate and develop their belief into a comprehensive system which may remain unaltered even with regular medication.

The multidimensionality of delusional experience also has implications for the conceptualization of the temporal course of psychotic decompensation and resolution. Individual dimensions of delusional experience often change independently of one another during the course of a psychotic episode, so that recovery can be determined by changes in one of the several dimensions (Garety and Freeman,1999).

## PATTERN OF RESOLUTION

- Encapsulation: Patients vary very much in the degree to which they can maintain their original personality and adapt to a normal life. It is frequently seen in residual states.
- In some cases one sees a longitudinal splitting as it were in the current of life, both the reality adapted and the delusional life go on alongside each other.
- On certain occasions (e.g. Meeting certain people, return to familiar locations, meeting the doctor who had treated the patient) the delusional complex comes to the surface and florid symptoms reappear.

Jorgensen (1995) found three types of recovery, one with full and the other two with partial recovery of delusional beliefs. In patients with partial recovery, decrease in pressure precede, decrease in other dimensions. For two-thirds there was no change in the degree or insight during recovery.

## CONCLUSION

Delusions are a key clinical manifestation of psychosis and have particular significance for the diagnosis of schizophrenia. Although common in several psychiatric conditions, they also occur in a diverse range of other disorders (including brain injury, intoxication and somatic illness). Delusions are significant precisely because they make sense for the believer and are held to be evidentially true, often making them resistant to change. Although an important element of psychiatric diagnosis, delusions have yet to be adequately defined. The last decade has witnessed a particular intensification of research on delusions, with cognitive neuroscience-based approaches providing increasingly useful and testable frameworks from which to construct a better understanding of how cognitive and neural systems are involved. There is now considerable evidence for reasoning, attention, metacognition and attribution biases in delusional patients. Recently, these findings have been incorporated into a number of cognitive models that aim to explain delusion formation, maintenance and content. Although delusions are commonly conceptualized as beliefs, not all models make reference to models of normal belief formation. It has been argued that aberrant prediction error signals may be important not only for delusion formation but also for delusion maintenance since they drive the retrieval and reconsolidation-based strengthening of delusional beliefs, even in situations when extinction learning ought to dominate. Given the proposed function of reconsolidation, in driving automaticity of behavior it is argued that in an aberrant prediction error system, delusional beliefs rapidly become inflexible habits. Taking this translational approach will enhance our understanding of psychotic symptoms and may move us closer to the consilience between the biology and phenomenology of delusions.
